# Complete Genome Sequence of a Bacteroides fragilis Bacteriophage, vB_BfrS_NCTC

**DOI:** 10.1128/MRA.00548-21

**Published:** 2021-07-22

**Authors:** Mohammad A. Tariq, Simon R. Carding

**Affiliations:** a Gut Microbes and Health Research Programme, Quadram Institute Bioscience, Norwich, United Kingdom; b Norwich Medical School, University of East Anglia, Norwich, United Kingdom; University of Maryland School of Medicine

## Abstract

Bacteroides fragilis is an obligate anaerobe and a common gut commensal bacterium that is also an important opportunistic pathogen. Here, we present the complete genome sequence of the circularly permuted B. fragilis bacteriophage vB_BfrS_NCTC. It comprises 47,160 bp, with 69 open reading frames.

## ANNOUNCEMENT

Bacteroides fragilis is a constituent of the human colonic microbiota that, as a result of disruptions due to, for example, infections or antibiotic exposure, can gain access to the systemic circulation, leading to severe and sometimes fatal disease (1). It is inherently resistant to many antibiotics, and the rise in antimicrobial resistance has led to a renewed interest in phage therapy (2). We have isolated a novel bacteriophage, vB_BfrS_NCTC, and provide its complete genome sequence here.

The bacteriophage was isolated from samples obtained from a sewage wastewater treatment facility in the United Kingdom; sewage filtrate was screened against B. fragilis NCTC 9343. A 100-ml aliquot of wastewater was filtered through a 0.2-μm polyethersulfone (PES) syringe filter (Minisart; Sartorius), and the filtrate was concentrated using a 100-kDa cellulose centrifugal filter (Amicon Ultra-15; Millipore). The concentrated sample was resuspended in phosphate-buffered saline (PBS) to 1,500 μl. This sewage concentrate was added to 5 ml semisoft agar (0.35% [wt/vol]) containing 200 μl B. fragilis NCTC 9343 in mid-exponential growth phase. The plate was incubated for 16 h in an anaerobic cabinet, and resulting single plaques were purified an additional three times before propagation using the *Bacteroides* phage recovery medium (BPRM) top agar method (3). In brief, purified phage lysate was mixed with 200 μl mid-log-growth-phase bacterial culture in semisoft BPRM agar (0.35% [wt/vol]) and incubated for 16 h in an anaerobic cabinet (5% CO_2_, 5% H_2_, and 90% N_2_ at 37°C and ∼25 lb/in^2^). SM buffer was used to harvest the lawn until the phage titer reached >10^9^ PFU/ml. The phage DNA was extracted using a phage kit (catalog number 46800; Norgen Biotek Corp.) according to the manufacturer’s instructions. The DNA was sequenced by the Quadram Institute Bioscience sequencing facility (Norwich, UK) on an Illumina NextSeq 500 system using the Nextera XT library preparation kit (2 × 150 cycles, v2 chemistry; Illumina, Saffron Walden, UK). For all further steps, standard settings were used unless stated otherwise. Paired-end sequencing reads were provided as FASTQ files. Adapters were removed using Trimmomatic v0.39 (4), and quality trimming was carried out using Sickle v1.33 at –q 30 and –l 15 (5). The read quality was assessed using FastQC v0.11.9 (6). These reads were *de novo* assembled using SPAdes v3.14.1 (7). The assembled contigs were determined to be circularly permuted and were confirmed with Circlator v1.5.5 using the Minimus2 circularization pipeline as an option (8). The reads were mapped back using BWA-MEM v0.7.17 (9) and SAMtools v1.12 (10), and coverage statistics were generated using Qualimap v2.2.1 (11). The genome open reading frames (ORFs) were predicted and initially annotated using the RAST server (12, 13). To comprehensively annotate the genome, ORFs were searched with BLASTp (14) against the NCBI nonredundant database (access date: 15 November 2020) at an E value of <1 e−5. ARAGORN was used to identify any tRNA genes (15).

The sequencing of vB_BfrS_NCTC yielded 1,012,042 clean reads, with a mean length of 129 bp. The reads had an average Phred quality score of 35, and the GC content was 39.56%. A total of 98.54% of the reads mapped back to the genome, giving a mean coverage depth of 2,793×. The assembled genome was shown to be circularly permuted at 47,160 bp, with a GC content of 38.83%. A total of 69 ORFs, with no tRNA genes, were identified. The genome comparison was performed using the default settings of the online tool BLASTN with the nonredundant/nucleotide database (16) and showed the greatest similarity at the nucleotide level to the B. fragilis phage Barc2635 (GenBank accession number MN078104), with 92% query coverage and 97% identity.

### Data availability.

The genome sequence of vB_BfrS_NCTC is available in GenBank under the accession number MW314138. The SRA data can be found under the accession number SRR13174062.
